# Potential Antimicrobial and Antibiofilm Properties of Copper Oxide Nanoparticles: Time-Kill Kinetic Essay and Ultrastructure of Pathogenic Bacterial Cells

**DOI:** 10.1007/s12010-022-04120-2

**Published:** 2022-09-10

**Authors:** Amr M. Shehabeldine, Basma H. Amin, Fatouh A. Hagras, Amr A. Ramadan, Mohamed R. Kamel, Mohamed A. Ahmed, Kareem H. Atia, Salem S. Salem

**Affiliations:** 1 Botany and Microbiology Department, Faculty of Science, Al-Azhar University, Nasr City, 11884 Cairo Egypt; 2 Regional Center for Mycology and Biotechnology (RCMB), Al-Azhar University, Nasr City, Cairo Egypt

**Keywords:** Copper oxide nanoparticles, Green synthesis, Anti-bacterial, Anti-biofilm activity

## Abstract

Mycosynthesis of nanoparticle (NP) production is a potential ecofriendly technology for large scale production. In the present study, copper oxide nanoparticles (CuONPs) have been synthesized from the live cell filtrate of the fungus *Penicillium chrysogenum*. The created CuONPs were characterized via several techniques, namely Fourier transform infrared (FTIR) spectroscopy, X-ray diffraction (XRD), transmission electron microscope (TEM), scanning electron microscope (SEM), and energy-dispersive X-ray spectroscopy (EDX). Furthermore, the biosynthesized CuONPs were performed against biofilm forming *Klebsiella oxytoca* ATCC 51,983, *Escherichia coli* ATCC 35,218, *Staphylococcus aureus* ATCC 25,923, and *Bacillus cereus* ATCC 11,778. The anti-bacterial activity result was shown with the zone of inhibition determined to be 14 ± 0.31 mm, 16 ± 0.53 mm, 11 ± 0.57 mm, and 10 ± 0.57 mm respectively. *Klebsiella oxytoca* and *Escherichia coli* were more susceptible to CuONPs with minimal inhibitory concentration (MIC) values 6.25 and 3.12 µg/mL, respectively, while for *Staphylococcus aureus* and *Bacillus cereus*, MIC value was 12.5 and 25 μg/mL, respectively. The minimum biofilm inhibition concentration (MBIC) result was more evident, that the CuONPs have excellent anti-biofilm activity at sub-MIC levels reducing biofilm formation by 49% and 59% against *Klebsiella oxytoca* and *Escherichia coli*, while the results indicated that the MBIC of CuONPs on *Bacillus cereus* and *Staphylococcus aureus* was higher than 200 μg/mL and 256 μg/mL, respectively, suggesting that these CuONPs could not inhibit mature formatted biofilm of *Bacillus cereus* and *Staphylococcus aureus* in vitro. Overall, all the results were clearly confirmed that the CuONPs have excellent anti-biofilm ability against *Klebsiella oxytoca* and *Escherichia coli*. The prepared CuONPs offer a smart approach for biomedical therapy of resistant microorganisms because of its promoted antimicrobial action, but only for specified purposes.

## Introduction

Antimicrobial resistance (AMR) is a worldwide threat to human health and progress. To attain the Sustainable Development Goals (SDGs), urgent multi-sectoral action is required [1]. Nanotechnology is becoming more widely used in medicine; thus, it is not surprising to find these technologies being used to combat the threat of antibiotic resistance. Nanoparticles can be used in a variety of ways to treat infections therapeutically [2–9]. They can be combined with currently available antimicrobials to improve their physiochemical action against drug-resistant bacteria [10, 11]. CuONPs can be made in a variety of ways, including physical, chemical, and biological processes [12–14]. Environmentally friendly biological approaches are favored above other alternatives. As a result, they are known as “green” methods. Plant extracts or microorganisms are the most common biological ways for reducing metal ions to metal NP [15–18]. Microbial methods are those that utilize microorganisms primary or secondary metabolites for metal oxide reduction [19–22]. According to the above statement, green synthesis methods have more economic, product purity, biocompatibility, and reaction required conditions than chemical synthetic methods [23–27]. Throughout time, studies have explored microbes inherent and adaptive drug-resistance capacities, focusing on changes in bacterial cell structure, mutations to inhibit drug and antibiotic targets, and actual modification and deactivation of bioactive compounds [28]. Biofilms are complicated populations of microbes that, owing to their adaptive character and durability, show bacterial resistance and the immune defense response [29]. Biofilm outbreaks are difficult to eradicate, especially when multi-drug resistance bacteria are involved. Biofilm-related disorders are often long-term infections with a gradual progression that can withstand both the immune system of the host and a transitory response to antimicrobial therapy [30–32]. Antibiotic therapy of biofilm communities has been found to be inefficient in recent years, as many medicines failed to reach its target cells deep within the biofilm matrix. To manage illness caused by biofilm communities, a new approach is required [33]. Nanoparticles are widely thought to be active and acceptable medications that can be used to increase the antibacterial activity of traditional antibiotics by combining these novel antimicrobials with key antibiotics in a synergistic combination therapy against pathogenic microorganisms [34, 35]. Herein, this study aimed to mycosynthesis of CuONPs from the culture supernatant broth of the filamentous fungus *Penicillium chrysogenum*. In addition, the characterization of mycosynthesized CuONPs will be assessed using FTIR spectroscopy, XRD, TEM, and SEM with EDX. Finally, evaluation of different antimicrobial activity, ultrastructure of pathogenic cells, and antibiofilm activities for mycosynthesized CuONPs will be investigated.

## Material and Methods

### Materials

Copper acetate monohydrate (Cu(CH_3_COO)_2_·2H_2_O) was purchased from Sigma-Aldrich for chemicals and used as precursors for CuO nanoparticles. Sodium hydroxide (NaOH) was obtained from Sigma-Aldrich (St. Louis, MO, USA) and dimethyl sulfoxide (DMSO) and trisodium citrate were supplied by Merck (Germany). Commercial antibiotics used in this study as a positive control such as a control antibiotic vancomycin for gram-positive organisms and colistin for gram-negative organisms were purchased from Liofilchem, Italy.

### Microbial Strains

Extended-spectrum beta-lactamase (ESBL) *Klebsiella oxytoca* ATCC 51,983, *Escherichia coli* ATCC 35,218, *Staphylococcus aureus* ATCC 25,923, *Bacillus cereus* ATCC 11,778, and *Penicillium chrysogenum* ATCC 48,271 were purchased from American Type Culture Collection (ATCC). In this study, the green synthesis of CuONPs was accomplished by filtrate of *Penicillium chrysogenum*.

### Synthesis of CuONPs by *Penicillium chrysogenum* Filtrate

*Penicillium chrysogenum* was grown up in a 250-mL Erlenmeyer flask, containing 100 mL potato-dextrose broth for 4 days at pH 6.5, 28 °C, and shaking of 120 rpm. The biomass of *P. chrysogenum* was separated using Whatman-paper No. 1 by filtration method. The filtrate of *P. chrysogenum* was used for CuONP formation as follows: 2 mM of copper acetate was mixed with filtrate of *Penicillium chrysogenum* and incubated for 4 h on the magnetic stirrer at 65 °C with 200 rpm. A green color appeared for CuONPs synthesis, at the end of reaction. The CuONPs were separated and dried at 100 °C for 24 h. The CuONP product was eventually collected and subjected for further investigation.

### Characterization of CuONPs

A variety of instrumental analytical methods were used to characterize the CuONPs. Using a Spectrum Two IR Spectrometer (PerkinElmer Inc., Shelton, USA) and these techniques, the total internal reflectance/Fourier-transform infrared (ATR-FTIR) spectra was used to semi-quantitatively measure the observable IR spectrum of the CuONPs by evaluating the transmittance over a spectral region of 4000 to 400 cm^−1^. To achieve a suitable signal quality, all spectra were collected at a 4-cm^−1^ resolution by collecting 32 scans. A Diano X-ray diffractometer (Philips) with a CuK radiation source (*λ* = 0.15418 nm) activated at 45 kV, as well as a generator (PW, 1930) and a goniometer (PW, 1820), was used to study the XRD pattern of the produced CuONPs. The shape and size of the prepared CuONPs were observed using the TEM method. The ultra-high resolution transmission electron microscope (JEOL-2010) with a voltage of 200 kV was employed. A drop of the particle solution was placed on a carbon-coated copper grid and dried under a light to create TEM grids. A field emission scanning electron microscope (SEM) installed with a field emission-gun (Quanta, 250-FEG) and connected with an energy-dispersive X-ray analyzer (EDX) with an excitation source of 30 kV for energy-dispersive X-ray evaluation (EDX) was used to examine the surfaces of the prepared CuONPs.

### In Vitro Antibacterial Activity and MIC Determination

The antibacterial activity of green CuONPs was tested in vitro using the agar well diffusion method [36]. For this study, two gram-negative bacteria *Klebsiella oxytoca* ATCC 51,983 and *Escherichia coli* ATCC 35,218 were used, as well as two gram-positive bacteria *Staphylococcus aureus* ATCC 25,923 and *Bacillus cereus* ATCC 11,778. Overnight cultures of each strain at 0.5 McFarland standard were spread onto Luria–Bertani (LB) plates, which were pierced with a 6-mm-diameter cork borer and loaded with 50 µL of CuONPs diluted in 1% DMSO at various concentrations (25, 50, and 100 μg/mL (w/v)). After incubation, the radius of the inhibition zone was measured with a Vernier caliper. Minimal inhibitory concentrations (MICs) against tested organisms were determined using the microbroth dilution method and resazurin dye [37, 38]. Micro-dilution of overnight grown culture strains (McFarland turbidity of 0.5) were cultured in 96-well plates using Luria–Bertani broth. Different concentrations of CuONPs (50, 25, 12.5, 6.25, 3.12, and 1.56 μg/mL) were added and plates were incubated at 37 °C for overnight. Then, 30 μL resazurin solution (0.1 mg/mL) was added to each well and incubated at 37 °C for at least 2 h. Any change in culture color from blue dye to pink within viable cells was assessed visually [39]. The lowest concentration of CuONPs in which the change in dye’s color occurred was taken as the MIC value. The MBC value was determined when no colony growth was observed after plating directly the contents of wells with concentrations higher than the MIC value [40]. MIC index (MBC/MIC) was used to describe antibacterial activity. Bactericidal activity is indicated by a MIC index of 1–2, on the other hand, MIC index 4–16 denotes the presence of bacteriostatic activity [41, 42]. A positive control of gentamicin was used.

### Time-Kill Kinetics

Time-kill kinetics assay of CuONPs was carried out as described by [43]. Briefly, overnight grown colonies of each strain were resuspended and incubated (37 °C, 180 rpm) for 2 h. An inoculum size of 1 × 10^6^ CFU/mL of each strain was inoculated into sterilized Luria–Bertani (LB) broth media, followed by addition of successive concentrations of CuONPs MIC (0.5 × MIC, 1 × MIC, 2 × MIC, 4 × MIC, and 8 × MIC), and then incubated (37 °C, 180 rpm). A growth control with no CuONPs (0.0 × MIC) was also included. Aliquots of 1 mL of cultures were taken at time intervals of 0, 15, 30, 60, and 120 h and were spread onto LB agar plates and incubated at 37 °C for 24 h. The viable cells were counted as CFU/mL.

### Morphological Observation by Transmission Electron Microscopy

Ultrastructure alterations of four tested bacterial strain treated with 2 × MIC from synthesized CuONPs at 37 °C for 24 h were examined using TEM. Bacterial cells were collected by centrifugation (at 4000 rpm for 10 min) from 24 h old cultures grown on nutrient broth media and washing with distilled water; the samples were fixed in 3% glutaraldehyde, rinsed in phosphate buffer, and post-fixed in potassium permanganate solution for 5 min at room temperature. The samples were dehydrated in an ethanol series ranging from 10 to 90% for 15 min in each alcohol dilution and finally with absolute ethanol for 30 min. Samples were infiltrated with epoxy resin and acetone through a graded series until finally in pure resin. Ultrathin sections were collected on copper grids. Sections were then double stained in uranyl acetate followed by lead citrate. Stained sections were observed with a JEOL-JEM 1010 transmission electron microscope at 70 kV at The Regional Center for Mycology and Biotechnology (RCMB), Al-Azhar University [44].

### Determination of Biofilm Inhibitory Concentration

A 96-well microtiter plate was used to determine the anti-biofilm activity of the CuONPs as described by [45, 46]. A two-fold dilution series of the CuONPs was used to determine the MIC of CuONPs against biofilm formation against test pathogen bacteria. Biofilm experiments were performed using static biofilm model and were determined by the crystal violet staining method. Biosynthesized CuONPs were diluted into 96-well plates as described above; six concentrations were diluted from 0.5 × MIC. The plates were incubated under aerobic conditions at 37 °C for 48 h discarding the liquid mixture, and the wells were stained with 0.1 mL 0.4% crystal violet for 15 min after being washed with sterile water twice. Then, samples were rinsed with distilled water twice and the dye bound to biofilm was solubilized by adding ethanol (95%). Absorbance of the isolated dye was measured quantitatively at 540 nm. All trials were performed in duplicate to reduce the error rate as much as possible.

### Statistical Analysis

GraphPad Prism 8.0 (software 2019, San Diego, CA, USA) was used to analyze all results. All data were represented as means ± standard deviation from at least three independent experiments (*n* = 3). ANOVA and Tukey’s multiple comparisons test were used to analyze the significant difference between all groups’ results. *P* < 0.05 was considered statistically significant.

## Result and Discussion

### Biosynthesis and Characterization of CuONPs

**Fig. 1:**
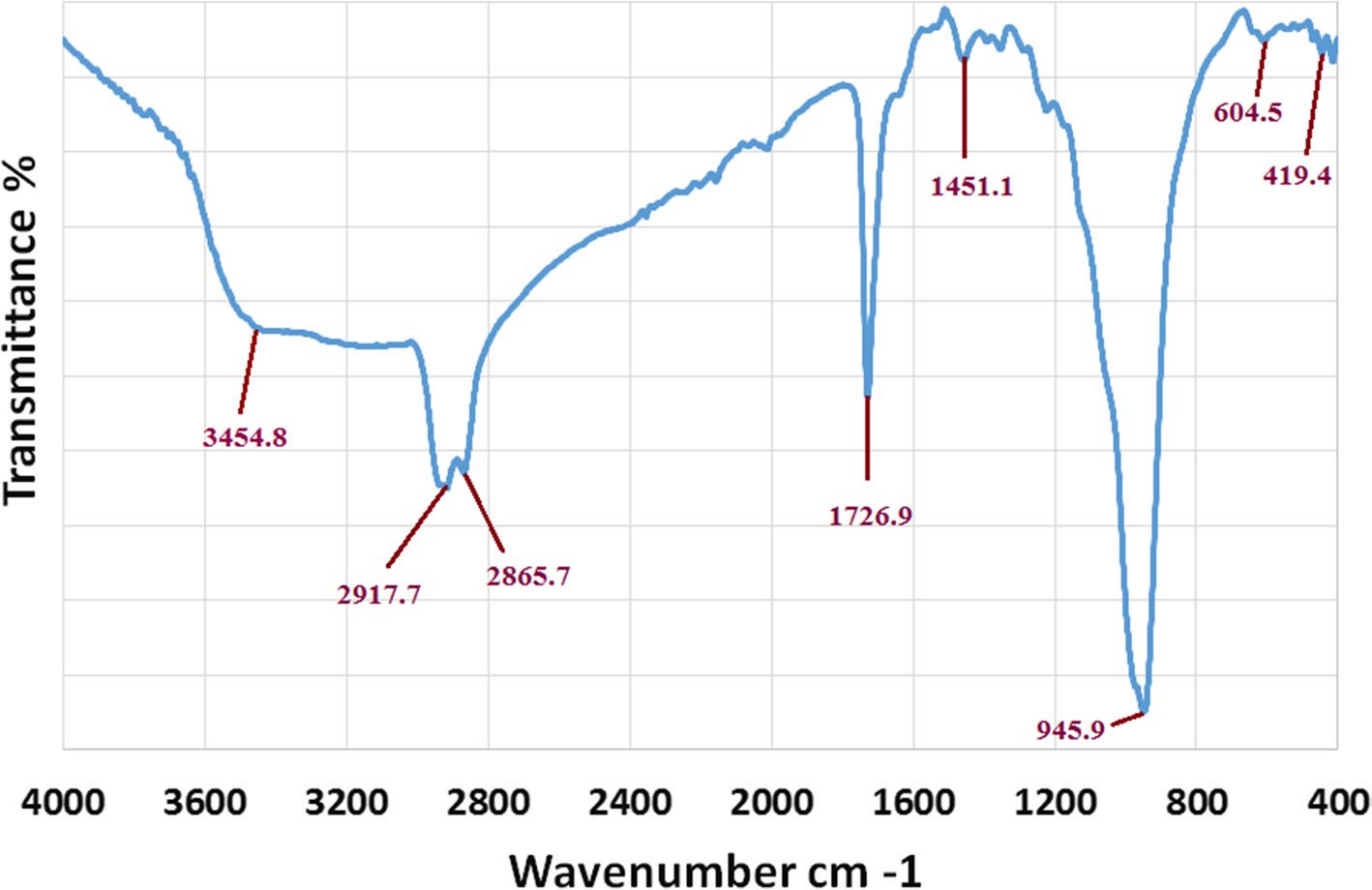
FTIR spectrum of CuONPs synthesized by *P. chrysogenum* filtrate

The use of biological materials in the green production of metal oxide nanoparticles has recently piqued researchers’ interest as a substitute to physical and chemical procedures. Different microorganisms and plants release metabolites that aid in the reduction, capping, and stabilization of CuO to CuONPs. The purpose of this research was to employ *P. chrysogenum* filtrate to produce CuO-nanoparticles. The fundamental aim of this strategy is to present a clean and ecofriendly way to make CuO-nanoparticles utilizing *P. chrysogenum* filtrate. CuONPs were prepared using copper acetate as a precursor, which was added to the *P. chrysogenum* filtrate until a gradual change in reaction color was detected. The combination alters the color of the solution to green, indicating that CuONPs are formed by the *P. chrysogenum* filtrate [47]. FTIR spectroscopic research was also carried out to validate the possible function of *P. chrysogenum* filtrate in CuONPs biosynthesis. By detecting the excitations of chemical bonds, FTIR can determine the functional groups that are present on the surface of CuO-nanoparticles. The interaction of a capping agent from *P. chrysogenum* filtrate with CuONPs is indicated by wave numbers at 3454.8 cm^−1^, 2917.7 cm^−1^, 2865.7 cm^−1^, 1726.9 cm^−1^, 1451.1 cm^−1^, 945.9 cm^−1^, 604.5 cm^−1^, and 419.4 cm^−1^ in Fig. 1. The lines in the spectra at 3454.8 cm^−1^ correspond to O–H stretch vibrations, suggesting that *P. chrysogenum* filtrate contains alcohol and phenol groups [48]. The alkane-CH group is responsible for the bands at 2917.3 cm^−1^ and 2865.7 cm^−1^. The existence of C-O of the ester group is indicated by the strong peaks seen on 1726.9 cm^−1^. The existence of N–H amine is shown by the peaks at 1451.1 cm^−1^. The peak at 945.9 cm^−1^ is attributed to the C–C- bending of alkenes [49]. The FTIR sections of the spectra of CuONPs showed peaks at 604.5 cm^−1^ and 419.4 cm^−1^, which were attributed to the binding of CuONPs with bio-materials prepared by *P. chrysogenum* filtrate [47]. Carbohydrates and proteins were shown to be the most abundant on the surface of *P. chrysogenum* filtrate, according to FTIR analyses. The peaks’ variations suggest that organic constituents in the filtrate of *P. chrysogenum* have successfully supported the formation of CuONPs via the reduction process, and might help protect CuONPs from aggregating and hence maintain their long-term stability.

**Fig. 2:**
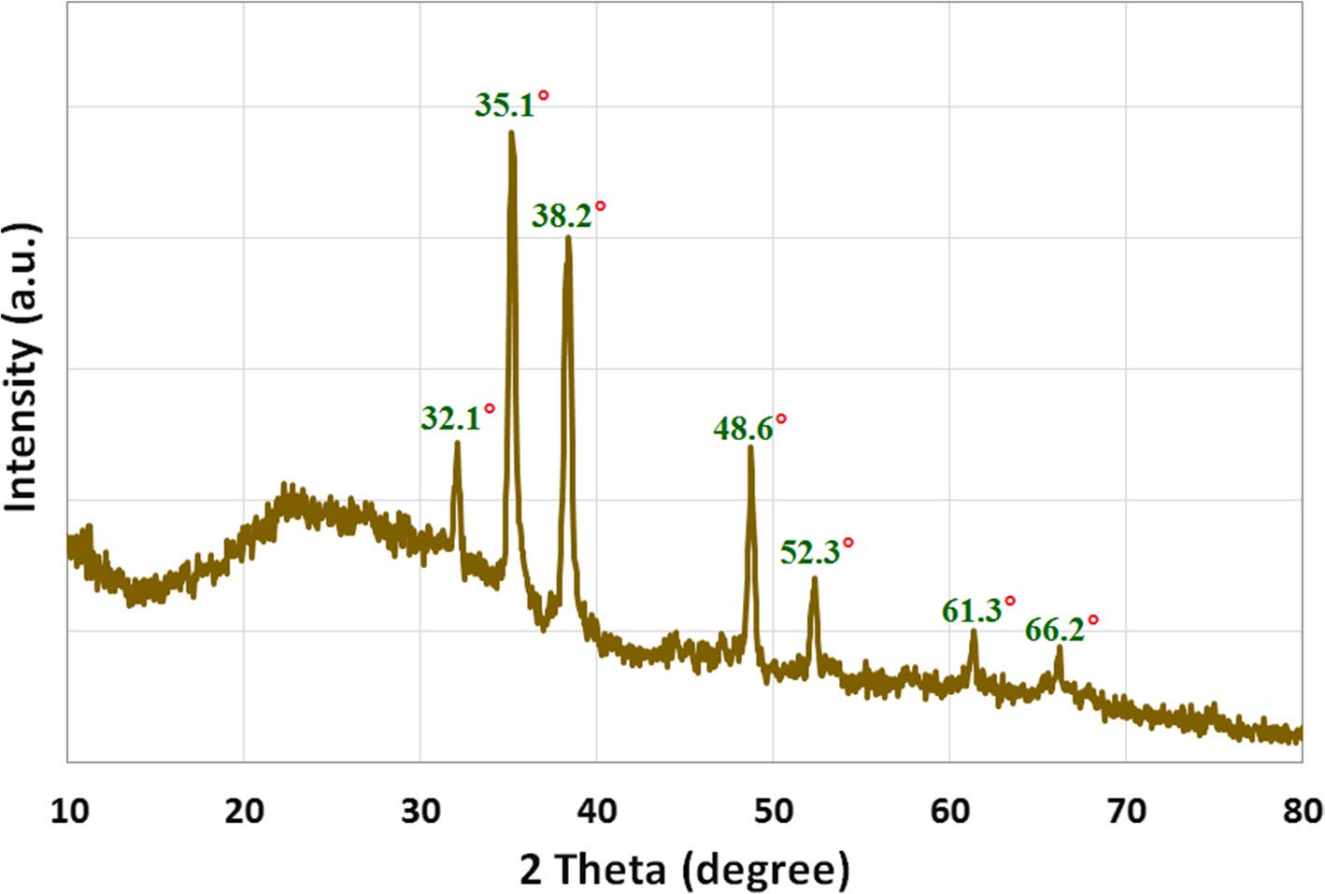
XRD pattern of CuONPs synthesized by *P. chrysogenum* filtrate

The crystalline structure of CuONPs was validated using XRD analysis, as shown in Fig. 2A. The primary strong angles in the diffractogram of bio-synthesized CuONPs were visible in the XRD patterns, showing that CuONPs were crystallographic in nature. Figure 2A shows XRD diffraction peaks of CuONPs, and displayed the diffraction characteristics regarding 2θ at 32.1°, 35.1°, 38.2°, 48.6°, 52.3°, 61.3°, and 66.2°, which represented Bragg’s reflections at 110, − 111, 111, − 20, 020, − 113, and 022 respectively. All the peaks were similar to the Joint Committee on Powder Diffraction Standards (JCPDS) of CuONPs with a standard card JCPDS file No: 01–1117 [50]. Therefore, the results clearly support the CuONP synthesis. The CuONP diffractogram does not reveal the existence of any other impurities. The findings of the present investigation were consistent with those of Badawy et al. [47] who found the similar monoclinc diffraction pattern for CuONPs. It ensures that the CuONPs obtained are pure.

**Fig. 3:**
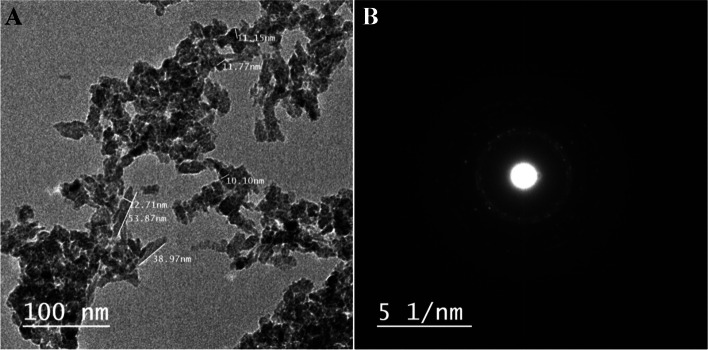
TEM image (**A**) and SAED pattern (**B**) of the synthesized CuONPs by *P. chrysogenum* filtrate

The most widely used technique for determining the morphological features and sizes of produced nanostructures is the transmission-electron microscope (TEM). The CuONPs are created in various forms such as rod and spherical with average size ranges of 11–53.8 nm and 4–15 nm, respectively, as seen in the TEM picture (Fig. 3A). The planes of CuONPs, as well as the degree of crystallinity of *P. chrysogenum* filtrate CuO-nanoparticles, were shown by the bright circular areas in the SAED pattern (Fig. 3B). According to Saravanakumar et al. [51], *Trichoderma asperellum* produced CuONPs with a particle size ranging from 10 to 190 nm and a nearly spherical shape. Other studies demonstrated different sizes and forms of CuONPs produced by *Purpureocillium lilacinum*, *Aspergillus terreus*, *P. chrysogenum*, and *Aspergillus niger* [36, 47, 50, 52].

**Fig. 4:**
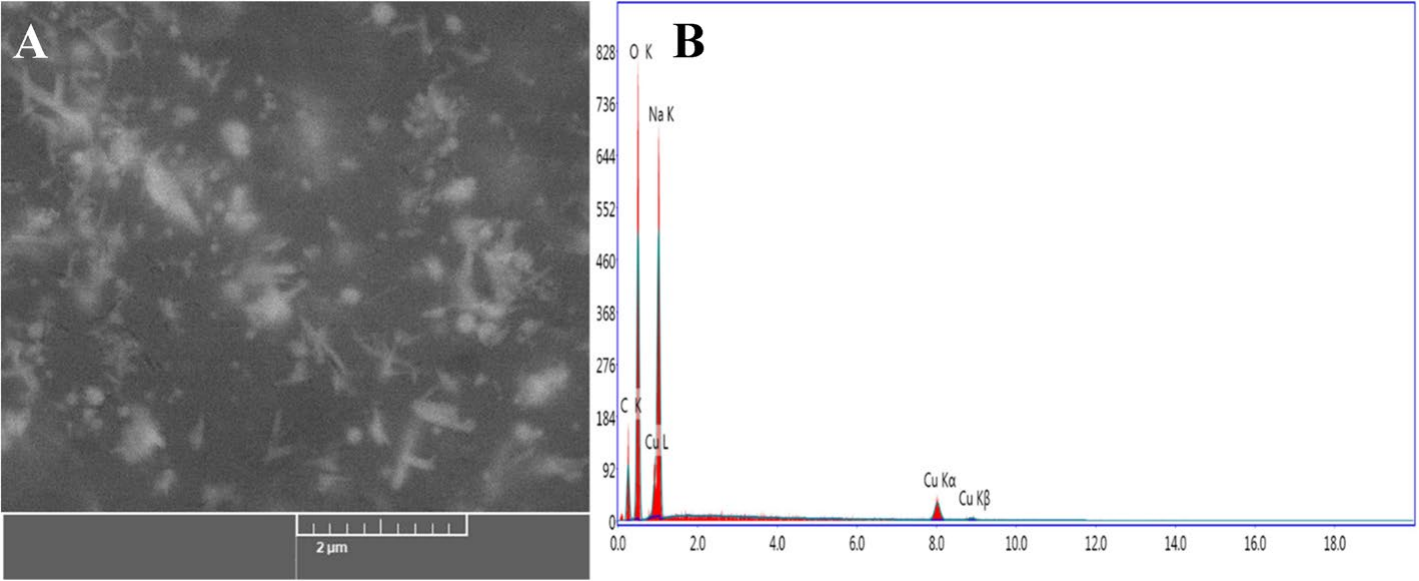
**A**) SEM image and **B**) EDX spectrum of the synthesized CuONPs by *P. chrysogenum* filtrate

As shown in Fig. 4A, the SEM was used to evaluate the surface morphology and particle-size of CuONPs [36, 52]. CuONPs had a form that was virtually rod and spherical. EDX analysis was used to determine the elemental composition of the CuONPs powder. In the CuONPs, the EDX spectra revealed the existence of several well-defined bands associated to copper (Cu), oxygen (O), and carbon (C) components (Fig. 4B). The carbon (C) signal comes from the metabolites of *P. chrysogenum* filtrate, whereas the copper (Cu) and oxygen (O) peaks indicate the creation of CuO-nanostructures. Furthermore, EDX spectra revealed the generation of very pure CuONPs with no additional impurity-related peaks. According to Hassan et al. [48], Cu and O were the main EDX spectrum peaks for CuONPs produced by microorganisms, with the existence of additional minor peaks relating to biomolecules in the microorganisms filtrate that linked with CuONPs.

### Determination of Antimicrobial Activity of Biosynthesized CuO-NPs

**Fig. 5:**
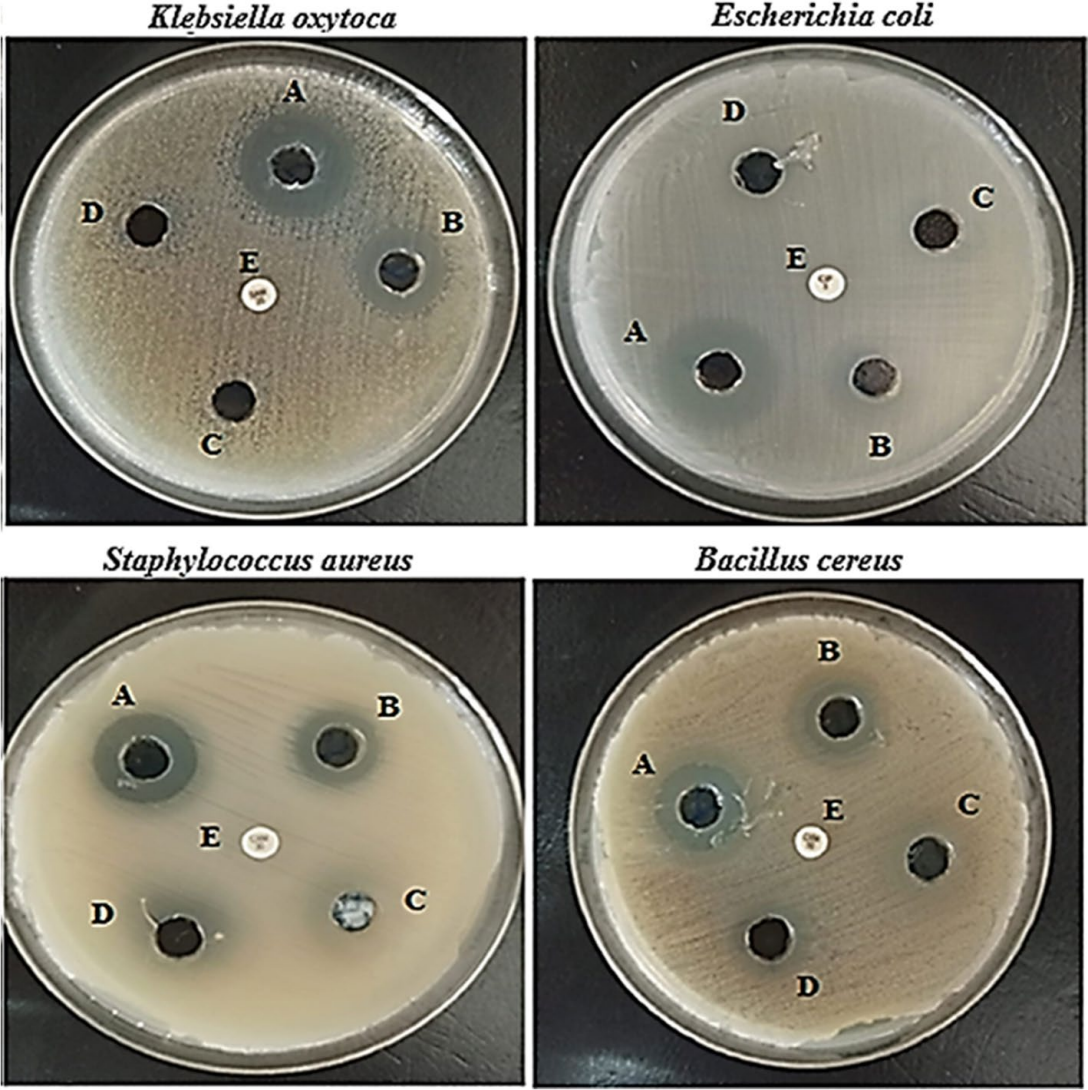
Antimicrobial activity of different concentration of CuONPs (**A**, 100 µg/mL; **B**, 50 µg/mL; **C**, 25 µg/mL; **D**, 12.5 µg/mL) as indicated by growth-inhibition zone of CuONPs against *Klebsiella oxytoca*, *Escherichia coli*, *Staphylococcus aureus*, and *Bacillus cereus*. Control antibiotic vancomycin for gram-positive organisms and colistin for gram-negative organisms

Agar diffusion test for assessing the antibacterial activity of CuONP showed a clear zone of antimicrobial activity against *Klebsiella oxytoca* ATCC 51,983, *Escherichia coli* ATCC 35,218, *Staphylococcus aureus* ATCC 25,923, and *Bacillus cereus* ATCC 11,778. The diameters of inhibition zones are summarized in Table 1 and illustrated in Fig. 5. The results showed that when the concentration of nanoparticles increases the zone of inhibition grows. In *Klebsiella oxytoca*, *Escherichia coli*, *Staphylococcus aureus*, and *Bacillus cereus*, the zone of inhibition was determined to be 14 ± 0.31 mm, 16 ± 0.53 mm, 11 ± 0.57 mm, and 10 ± 0.57 mm, respectively (Fig. 5a–d). CuONPs that have been synthesized have the potential to be particularly effective against a variety of bacteria strains [53]. The distinctive high surface to volume ratio of copper nanoparticles permits them to interact with the cell membrane of the bacteria through its surface, resulting in the death of the bacteria [54]. CuONPs prevent the growth of microbes, which has a bactericidal effect. CuONPs with a small size and a large surface area cause electronic interactions, which is useful for improving the surface reactivity of NPs. Furthermore, the increased surface area percent immediately interacted with the bacterium, resulting in improved bacteria interaction. The antibacterial activity of NPs with a large surface area was greatly increased by these two critical features [55].

**Table 1:** Antimicrobial activity as indicated by growth-inhibition zone of different concentration of CuONPs against different strains of bacteria

Bacteria strains	Inhibition zone (mm)			
	Different conc. of CuONPs			
	100 µg/mL	50 µg/mL	25 µg/mL	12.5 µg/mL
*Klebsiella oxytoca* ATCC 51,983	14 ± 0.31	11 ± 0.24	ND	ND
*Escherichia coli* ATCC 35,218	16 ± 0.53	12 ± 0.17	ND	ND
*Staphylococcus aureus* ATCC 25,923	11 ± 0.57	9 ± 0.1	8 ± 0.17	7 ± 0.57
*Bacillus cereus* ATCC 11,778	10 ± 0.57	9 ± 0.1	7 ± 0.17	7 ± 0.57

The antimicrobial activity of CuONPs against model strains of *Klebsiella oxytoca*, *Escherichia coli*, *Staphylococcus aureus*, and *Bacillus cereus* has been investigated and was performed by microdilution assays using a resazurin-based microtiter dilution assay (RMDA) method. The advantages of this assay in detection of antibacterial assays include increased sensitivity and ability to distinguish between bacteriostatic and bactericidal effects [56]. In the resazurin-based assay, all sterility control wells remained blue after incubation for 24 h. In contrast, all wells which containing tested microorganisms changed from blue to pink indicating normal growth. The result revealed that both gram-positive and gram-negative bacteria tested were susceptible to CuONPs. *Klebsiella oxytoca* and *Escherichia coli* were more susceptible to CuONPs with MIC values 6.25 and 3.12 µg/mL, respectively, while for *Staphylococcus aureus* and *Bacillus cereus*, MIC value was 12.5 and 25 μg/mL, respectively (Table 2). Our findings showed that the CuONPs have a better antibacterial effect against gram-negative bacteria. This could be because gram-positive bacteria has a strong cell wall, whereas gram-negative bacteria has a thin cell wall so the CuONPs easily penetrate to the cell membrane of the gram-negative bacteria and cause damages to the cell [57]. The MBC is the minimum concentration of biosynthesized CuONPs which is required to kill the bacterium completely under specific conditions (showed no growth on the agar plate) [58]. The MBC value was determined as the lowest concentration at which there was no bacterial growth. MBC values of the CuONPs after 24 h were ranged from 12.5 to 50 µg/mL. The lowest MBC value of CuONPs was 12.5 and 25 µg/mL, inferring that it can kill more than 99% of *Klebsiella oxytoca* and *Escherichia coli* respectively. In contrast, the concentration necessitated for CuONPs to achieve the similar effect on *Staphylococcus aureus* and *Bacillus cereus* was 50 µg/mL. Antibacterial agents are usually regarded as bactericidal if the MBC/MIC ratio is ≤ 4 and bacteriostatic if > 4 [59]. The MBC/MIC ratios of CuONPs were equal to 4 for *Klebsiella oxytoca*, *Escherichia coli*, and *Staphylococcus aureus* strains, indicating bactericidal activity while MBC/MIC ratios of CuONPs were equal to 2 for *Bacillus cereus* which indicating bacteriostatic effect.

**Table 2:** The MIC values determined by colorimetric assay (resazurin), MBC (99.9% kill) and MIC/MBC ratio of mycosynthesized CuONPs against bacterial strains

Bacterial strains	MIC (μg/mL)	MBC (μg/mL)	MBC/MIC ratio	Control antibiotic^a^ (μg/mL)
Klebsiella oxytoca ATCC 51,983	6.25	25	4	5
Escherichia coli ATCC 35,218	3.12	12.5	4	5
Staphylococcus aureus ATCC 25,923	12.5	50	4	7.5
Bacillus cereus ATCC 11,778	25	50	2	10

^a^Control antibiotic: vancomycin for gram-positive bacteria and colistin for gram-negative bacteria

### In Vitro Susceptibility Testing and Time-Kill Kinetic Assay

**Fig. 6:**
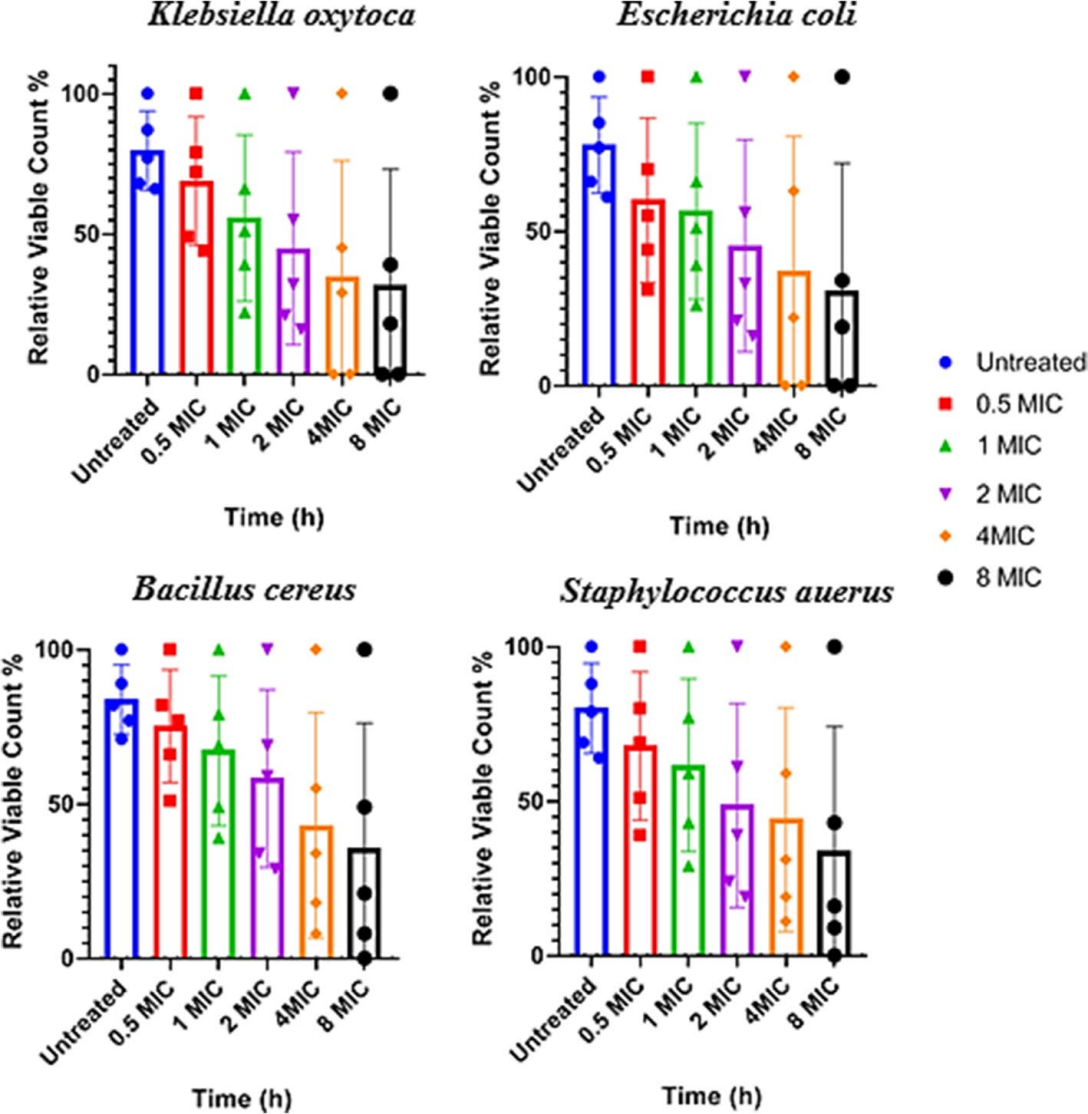
Time-kill plots of CuONPs against human pathogenic bacterial strains: *Klebsiella oxytoca*, *Escherichia coli*, *Bacillus cereus*, and *Staphylococcus aureus* at different concentrations and time-length. Each point represents the relative viable count of bacterial cells at a particular time intervals

This reduction was dependent on time and CuONP concentrations (Fig. 6). The relative of viable count for *Escherichia coli* was reduced by 56% at 2 × MIC, 63% at 4 × MIC, and 34% at 8 × MIC of CuONPs at 2 h.; while for *Klebsiella oxytoca*, it was reduced by 55% at 2 × MIC, 45% at 4 × MIC, and 39% at 8 × MIC of CuONPs at 2 h. The bactericidal endpoint of CuONPs for *Escherichia coli* was reached after 6 and 8 h of incubation with 4 × MIC (12.48 µg/mL) and 8 × MIC (24.96 µg/mL). The bactericidal endpoint of CuONPs for *Klebsiella oxytoca and Escherichia coli* was reached after 6 and 8 h of incubation with 4 × MIC (25 µg/mL) and 8 × MIC (50 µg/mL) respectively. The relative of viable count for *Bacillus cereus* was reduced by 69% at 2 × MIC, 55% at 4 × MIC, and 49% at 8 × MIC of CuONPs at 2 h. Similar observations were observed for *S. aureus*. The relative of viable count for *S. aureus* was reduced by 61% at 2 × MIC, 59% at 4 × MIC, and 43% at 8 × MIC of CuONPs at 2 h, also the end-point reached for both *S. aureus* and *B. cereus* faster after at (8 × MIC) 100 and 200 µg/mL, respectively, no significant differences (*P* > 0.05) were found among the two tested bacteria. Overall, results showed broad spectrum activity of CuONPs against the tested bacteria. Green synthesized CuONPs consider potent antibacterial agents due to their strong biocidal effect against different tested microorganism. Because gram-negative bacteria have weaker cell walls than gram-positive bacteria, they were damaged in a short period and at low concentrations when compared to typical compounds with antibacterial capabilities. This could be due to the increased surface area and positive surface density, which allows for better interaction with negatively charged bacterial cell membranes, as well as an increase in cell permeability and penetration of nano-sized particles into the bacterial cell, resulting in bacterial cell death [60, 61].

### Ultrastructure of Tested Bacterial Cells with Pretreated CuONPs

**Fig. 7:**
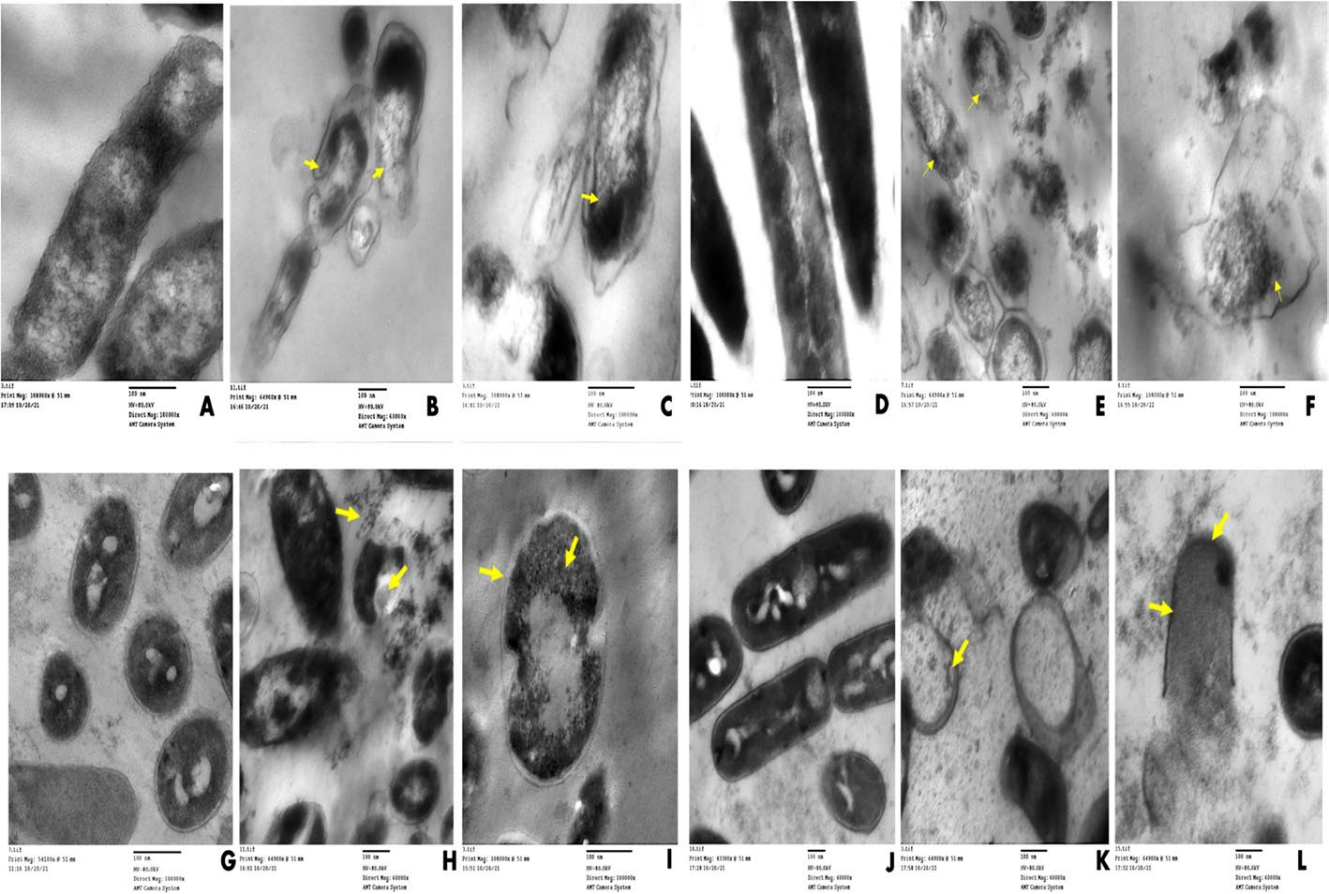
Transmission electron microscopy images of bacterial strains treated with CuONPs. *Escherichia coli* (**A**) control; (**B** and **C**) *Escherichia coli* affected by CuONPs (2 × MIC); *Bacillus cereus* (**D**) control; (**E** and **F**) *Bacillus cereus* affected by CuONPs (2 × MIC); *Staphylococcus aureus* (**G**) control; (**H** and **I**) *Staphylococcus aureus* affected by CuONPs (2 × MIC); *Klebsiella oxytoca* (**J**) control; *Klebsiella oxytoca* (**K** and **L**) affected by CuONPs (2 × MIC); scale bar = 100 nm

To demonstrate the interaction of CuONPs with tested bacteria strains and to capture various events of cellular damage, TEM studies were performed. The antimicrobial mechanism of CuONPs was investigated by the treatment of tested microbial pathogen cells with 2 × MIC of CuONPs in the PDA (liquid medium) for 48 h. TEM analysis revealed that cells in control cultures and cells exposed to CuONPs did not show severe damage, such as the formation of pits or the rupture of the cell wall (Fig. 7). On the other hand, CuONPs were found in most cases surrounding the examined cells (Fig. 7). All TEM ultrastructure bacterial cells treated with copper nanoparticles showed a widening periplasmic gap and extensive cytoplasmic swelling was observed compared with control cells. Ultrastructure of *Escherichia coli* untreated control rod cells with normal morphology was arranged in an ordered way (Fig. 7A). According to Figure 7B and C, *Escherichia coli* exposed to 2 × MIC CuONPs had changes in bacterial morphology, cell wall rupture, and a decrease in electron density in addition to a large concentration of very small particles in the cell wall and part of the cytoplasm. Ultrastructure of *Bacillus cereus* untreated control rod cells with normal morphology was arranged in an ordered way (Fig. 7D); The affected *Bacillus cereus* cells with 2 × MIC of copper nanoparticles show that all cells were lysed and vacant of cytoplasmic fluid, with the cytoplasmic membrane totally shrinking (Fig. 7E, F), while alteration in cell structure of *Staphylococcus aureus* resulted in membrane damage that looked to have ruptured potentially with leakage of intracellular components, and cellular damage eventually leads to full cell deformation (Fig. 7H, I), respectively, compared to control cell which showed a spherical-shaped structure with an undamaged and intact outer membrane (Fig. 7G). Ultrastructure changes when *Klebsiella pneumonia* is exposed with 2 × MIC CuONPs, and there were loss of smoothness and homogeneity in the cell membrane, as well as leakage of cytoplasmatic material with shrinkage and removal of cytoplasmic contents leads finally to completely cell deformation (Fig. 7K, L) compared with control cell that represented a unique and developed microstructure as shown in Fig. 7J. Inside the bacterial cytoplasm, smaller nanoparticles (arrows) were discovered. Finally, CuONPs broke the bacterial cell membrane, causing full cell lysis and the leaking of intracellular material. The precise method by which copper oxide inhibits the development of bacteria is still not completely known. There are, however, a number of explanations in the literature regarding the mechanism behind the antibacterial activity. First, the direct interaction mechanism: The bacterial cell can readily be harmed by the direct interaction of CuO with its surface [62]. In this instance, the bactericidal action is significantly influenced by the shape of the CuONPs [63]. Second, the bacterial molecules’ adherence to the metal ions released from nanoparticles [64, 65]. The bacterial cell membranes can eventually be damaged by copper ions because they are tiny enough to infiltrate the bacterial cells. As a result, the microbe with the negatively charged cell surface may make it easier for more copper ions to be captured by electrostatic attraction, which would lead to denaturation of protein and cellular damage [66]. Thirdly, the production of ROS such as superoxide anions, hydrogen peroxide, hydroxyl radicals, and organic hydroperoxides that can cause oxidative stress: the dissolution of CuO and eventual release of the copper ions can improve the ROS response and thereby significantly contribute to the destruction of the bacterial cell membrane [67].

### Anti-biofilm Evaluation

**Fig. 8:**
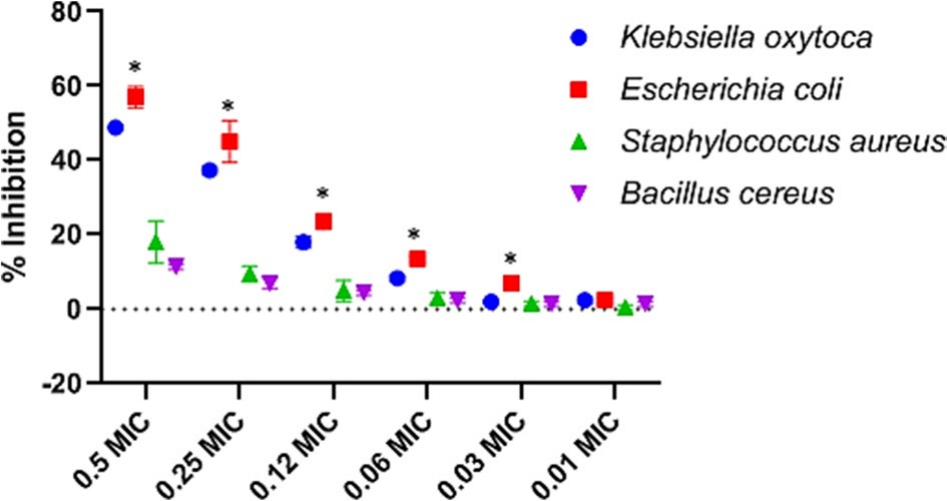
In vitro anti-biofilm activity of CuONPs at sub-MIC. The absorbance of the control was considered to represent 100% of biofilm (results were considered significant when compared to control; * *p* < 0.05)

The effects of CuONPs on bacterial biofilm development were investigated. CuONPs using 1% crystal violet staining and represented as a percentage, the inhibitory efficacy of the biofilm development was different after 24 h of CuONPs treatment. The anti-biofilm activity of the CuONPs employed in their manufacture is shown in Fig. 8 at various doses. CuONPs inhibited bacterial biofilms with considerable biofilm inhibitory efficacy (*p* < 0.05). Test bacterial pathogens demonstrated a concentration-dependent reduction in biofilm formation in a biofilm quantification technique (Fig. 8). The antibiofilm activity (minimum biofilm inhibition concentration, MBIC) of CuONPs against *Klebsiella oxytoca* and *Escherichia coli* at sub-MIC levels reduced biofilm formation by 49% and 59% at 0.5 × MIC, respectively. The results indicated that the MBIC of CuONPs on *Bacillus cereus* and *Staphylococcus aureus* was higher than 200 μg/mL and 256 μg/mL, respectively, suggesting that these CuONPs could not inhibit mature formatted biofilm of *Bacillus cereus* and *Staphylococcus aureus* in vitro. One of the most important reasons for the effect of CuONPs on biofilm inhibition is their particle size, as smaller particles have a larger surface area for interaction with microorganisms when compared to the bacterial control. By disrupting the bacterial cell wall or cell membrane, copper ions have the ability to limit bacterial growth and development. Because of their affinity for phosphorus and sulfur-rich molecules like DNA, they will interact with them once within the cell [68]. Anti-biofilm activities have been attributed to the capabilities of Cu^2+^ ions liberated from CuONPs, which fully immerse the bacterial cell surface and cause cell damage by altering structure of proteins and enzyme characteristics. CuONPs boost cellular enzyme activity, which improves cell permeability, which affects cell growth and biofilm development, depending on the physiochemical parameters of the growth medium [69].

## Conclusion

The broad spectrum applications of NPs are challenging researchers to explore the greatly effective, cost affordable, and eco-friendly methods for synthesis and develop the activity of NPs. Synthesis of CuONPs was performed via green method using *P. chrysogenum* filtrate. Furthermore, a promising effect of CuONPs appeared through antimicrobial activity as well as antibiofilm activity. Highly effective of CuONPs was observed against tested organisms including *B. cereus*, *S. aureus*, *E. coli*, and *K. oxytoca* compared to the effect of antibiotic. Furthermore, the CuONPs have excellent anti-biofilm ability against *K. oxytoca* and *E. coli*. In addition, morphological observation of tested bacterial strain after CuONPs internalization using TEM showed higher CuONPs found inside bacterial cells. Finally, this extracellular fungal synthesis of CuONPs has many advantages over the chemically derived nanoparticles and it could be concluded that CuONPs could be promising to reduce and enhance antibacterial action.

## Data Availability

The data used to support the findings of this study are available from the corresponding author upon request.
